# EMCMDA: predicting miRNA-disease associations via efficient matrix completion

**DOI:** 10.1038/s41598-024-63582-y

**Published:** 2024-06-04

**Authors:** Chao Qin, Jiancheng Zhang, Lingyu Ma

**Affiliations:** 1 School of Information Science and Engineering, Qilu Normal University, Jinan, 250200 China; 2 School of Control Science and Engineering, Harbin Institute of Technology, Weihai, 250200 China

**Keywords:** MiRNA-disease associations, Multi-source similarity, Heterogeneous information network, Truncated schatten p-norm, Matrix completion, Computational biology and bioinformatics, Biomedical engineering

## Abstract

Abundant researches have consistently illustrated the crucial role of microRNAs (miRNAs) in a wide array of essential biological processes. Furthermore, miRNAs have been validated as promising therapeutic targets for addressing complex diseases. Given the costly and time-consuming nature of traditional biological experimental validation methods, it is imperative to develop computational methods. In the work, we developed a novel approach named efficient matrix completion (EMCMDA) for predicting miRNA-disease associations. First, we calculated the similarities across multiple sources for miRNA/disease pairs and combined this information to create a holistic miRNA/disease similarity measure. Second, we utilized this biological information to create a heterogeneous network and established a target matrix derived from this network. Lastly, we framed the miRNA-disease association prediction issue as a low-rank matrix-complete issue that was addressed via minimizing matrix truncated schatten p-norm. Notably, we improved the conventional singular value contraction algorithm through using a weighted singular value contraction technique. This technique dynamically adjusts the degree of contraction based on the significance of each singular value, ensuring that the physical meaning of these singular values is fully considered. We evaluated the performance of EMCMDA by applying two distinct cross-validation experiments on two diverse databases, and the outcomes were statistically significant. In addition, we executed comprehensive case studies on two prevalent human diseases, namely lung cancer and breast cancer. Following prediction and multiple validations, it was evident that EMCMDA proficiently forecasts previously undisclosed disease-related miRNAs. These results underscore the robustness and efficacy of EMCMDA in miRNA-disease association prediction.

## Introduction

MicroRNAs (miRNAs) is an RNA molecule of about 21 to 23 nucleotides in length, which is widely found in eukaryotes. Their primary function revolves around modulating gene expression at the translational level^1^. MiRNAs have a pivotal effect in diverse biological processes, encompassing cell differentiation, development, and metabolic regulation^2,3^. Furthermore, aberrant miRNA expression is intricately linked to the initiation and advancement of various diseases, encompassing cancer, immune system dysregulation, and metabolic disorders^4^. Therefore, miRNAs have received growing interest in the field of pharmacotherapy, becoming potential candidates for drug development^5^. Additionally, many studies has focused on exploring potential associations of miRNAs with diseases. For instance, high miR-21 levels are linked to shorter survival in individuals with squamous cell lung cancer, suggesting that it may be a potent biomarker^6^.

The discovery of potential miRNA-disease associations (MDAs) holds great promise for enhancing our understanding of disease mechanisms, identifying biomarkers, facilitating personalized therapies, and advancing the development of innovative drugs. With the exponential growth of biogenetic big data and the remarkable progress in artificial intelligence, a plethora of computational models are emerging as efficient alternatives to guide biological experiments^7–9^. In addition, the continuous development of interaction prediction studies across different areas of computational biology has brought profound insights into deciphering the intricate web of relationships between genetic markers, non-coding RNAs, and the onset and progression of diseases^10–15^. These advances have not only revealed the regulatory roles of genetic markers and ncRNAs but also highlighted their potential as therapeutic targets in a wide range of diseases.

In recent years, machine learning has gained widespread acceptance and produced impressive outcomes in the domain of MDA prediction. Ouyang et al.^16^ introduced a HGCLAMIR model. They combine integrated multi-view representation and hypergraph contrast learning techniques with view-aware attention mechanisms to forecast MDAs. Wang et al.^17^ presented a GAMCNMDF approach. They established an antagonistic matrix-complete network that interconnects miRNAs and diseases, which was subsequently indicated as a matrix. Li et al.^18^ proposed an innovative approach for MDA prediction, employing a combination of dichotomous network recommendation and the KATZ model (KATZBNRA). Xie et al.^19^ introduced a novel model known as WBNPMD. They initially established transfer weights by combining known biological similarities and meticulously equipped preliminary information. Subsequently, a two-step binary network algorithm was employed to predict MDAs. Dai et al.^20^ presented a cascade forest technique using multi-source data integration for MDA prediction (MDA-CF). They initially consolidated multi-source information correlated with diseases and miRNAs, and then employed autoencoder for dimensionality reduction. The MDA-CF model was subsequently utilized to predict MDAs.

Graph inference-based approaches for predicting MDAs have garnered significant attention in recent research. Wang et al.^21^ introduced the Meta-Subgraph-based Heterogeneous Graph Attention Network Model (MSHGATMDA). In their approach, they defined five distinct types of meta-subgraphs derived from known MDAs. This model can effectively extract features associated with MDAs, both within and across these meta-subgraphs, to predict previously unknown association relationships. Zhang et al.^22^ introduced a FLNSNLI approach, which relies on linear neighborhood similarity for network link inference. In this approach, known MDAs were transformed into dichotomous networks, with miRNAs/diseases represented as association maps. Subsequently, miRNA and disease similarities were computed using these association mappings, employing a rapid linear neighborhood similarity metric. A label propagation algorithm was then applied to score candidate MDAs, and FLNSNLI was predicted using a weighted average strategy. MDHGI^23^ derived predicted association probabilities using a sparse learning method based on matrix decomposition. Then, they constructed heterogeneous networks by incorporating the obtained biological information. Finally, they used this network information to acquire predictive scores.

Matrix completion, a viable approach employed in predicting MDAs, has garnered widespread recognition for its practicality and effectiveness. Chen et al.^24^ introduced a novel technique for forecasting MDAs using bounded nuclear norm regularization (BNNRMDA). Initially, they utilized valuable information on miRNAs and diseases to construct a diverse network. Then, a target matrix was defined using information from this network, and prediction was accomplished by minimizing the nuclear norm of this matrix. Xu et al.^25^ presented PMFMDA, a MDA prediction model using probability matrix decomposition. They utilized biological matrix information to create a probabilistic matrix decomposition model, resulting in a predictive scoring matrix. This matrix complemented the existing MDA matrix using available biomatrix information. Chen et al.^26^ introduced IMCMDA, a novel model grounded in induced matrix completion. This approach maximized the utilization of biological information to recover missing values within the correlation matrix. Chen et al.^27^ put forward NCMCMDA, a neighborhood constraint matrix completion model. All existing methods employ the nuclear norm as an alternative to rank. However, the nuclear norm disregards the physical interpretation of singular values, suffers from overshrinkage, and the approximations obtained are not precise.

In this research, we created a new and efficient matrix complement-based strategy to predict MDAs via minimizing matrix truncated schatten p-norm (EMCMDA). First, we calculated the similarities across multiple sources for miRNA/disease pairs and combined this information to create a holistic miRNA/disease similarity measure. Second, we utilized this biological information to create a heterogeneous network and established a target matrix derived from this network. Lastly, we framed the MDA prediction issue as a low-rank matrix-complete issue that was addressed by minimizing matrix truncated schatten p-norm. The primary contributions of this work are outlined below.

1. We calculated the similarities across multiple sources for miRNA/disease pairs and combined this information to create a holistic miRNA/disease similarity measure. This enriches the similarity types, reduces the bias caused by a single similarity, and improves the similarity accuracy of miRNA/disease.
2. We used the truncated schatten p-norm minimization approach to complement the predicted scores for the unknown MDAs. The truncated schatten p-norm offers a more accurate estimation of the rank than other rank relaxation norms, and therefore obtains more accurate solutions. Furthermore, we have replaced the conventional singular value contraction algorithm with a weighted singular value contraction technique. This technique dynamically adjusts the degree of contraction based on the significance of each singular value, ensuring that the physical meaning of these singular values is fully considered.
3. The results from both Global LOOCV and 5-fold CV using the benchmark dataset clearly show that EMCMDA exceeds the the area under the ROC curve (AUC) of all compared methods. When applied to the HMDD v3.0 dataset, EMCMDA yielded impressive AUCs of 0.9725 and 0.9706 based on Global LOOCV and 5-fold CV, respectively. These findings underscore EMCMDA’s robust generalization capacity across diverse datasets. Furthermore, we implemented two case studies to illustrate the practical efficacy of EMCMDA.

**Figure 1:**
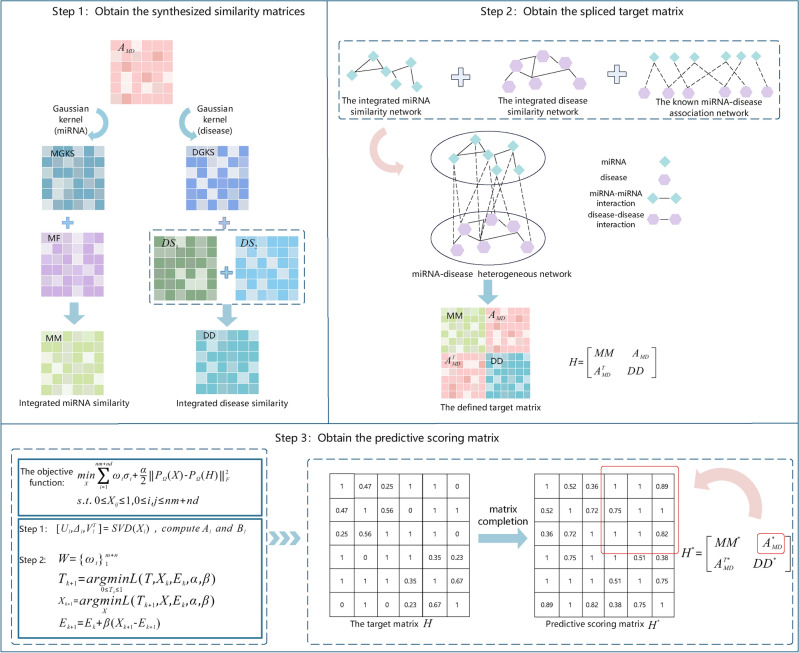
The framework of EMCMDA. Step1, computing and integrating miRNA/disease multi-source similarities to obtain a comprehensive miRNA/disease similarity; Step2, building a heterogeneous network and creating a target matrix derived from that network; Step3, minimizing the matrix truncated schatten p-norm and using a weighted singular value contraction algorithm yields the predicted score matrix.

## Materials and methods

The EMCMDA model is structured around three key phases, as illustrated in Fig. 1. First, we calculated the similarities across multiple sources for miRNA/disease pairs and combined this information to create a holistic miRNA/disease similarity measure. Second, we utilized the preprocessed data to create a heterogeneous network and established a target matrix derived from this network. Third, we complemented the missing values of the correlation matrix by minimizing matrix truncated schatten p-norm.

### Human MDAs

In this study, we employed a dataset comprising 5430 human-related MDAs, involving 383 diseases and 495 miRNAs, sourced from the HMDD v2.0 database^28^. This collection of biological data is herein referred to as the benchmark dataset. We constructed an association matrix $A_{MD}\in R^{nm \times nd }$ to represent known MDAs. Here, *nd* and *nm* denoted the respective counts of diseases and miRNAs. If miRNA $m_{i}$ is related to disease $d_{j}$, the corresponding element $A_{MD} (m_{i},d_{j})$ is assigned the value 1; otherwise, it is set to 0. The construction of the association matrix proceeded as shown below:

1 $$\begin{aligned} A_{MD}(m_{i}, d_{j})&= \left\{ \begin{array}{ll} 1 &{} \text{ if } m_{i} \text{ and } d_{j} \text{ have } \text{ association } \\ 0 &{} \text{ otherwise } \end{array}\right. \end{aligned}$$

### MiRNA functional similarity

Considering the observation that resemble miRNAs are frequently linked to resemble diseases, we acquired miRNA functional similarity. The data on miRNA functional similarity can be accessed from the link http://www.cuilab.cn/files/images/cuilab/misim.zip, as introduced by Wang et al.^29^. We created a matrix denoted as $MF\in R^{nm\times nm }$, which served to denote the data. The values contained in this matrix, represented as $MF(m_{i},m_{j} )$, fall within the range [0,1], reflecting the similarity level between miRNA $m_{i}$ and $m_{j}$.

### Disease semantic similarity

In this research, we integrated two methods for computing disease semantic similarity to improve accuracy. First, we compute the semantic correlation of each disease node by different methods, which in turn leads to Disease Semantic Similarity 1 and Disease Semantic Similarity 2. Subsequently, by integrating these two semantic metrics and applying weighted averaging, we obtain a comprehensive disease similarity metric. This integrated approach not only enriches the computational process, but also greatly improves the accuracy of disease similarity assessment.

#### Disease semantic similarity 1

Wang et al.^29^ introduced a approach for assessing semantic similarity in diseases by utilizing Medical Subject Headings (MeSH). For disease *d*, they built a directed acyclic graph labeled as $DAG_{d}$. The graph consists of three parts, specifically including the ancestor node *d*, *d* itself, and the direct edges connecting the parent node to its respective children.

In $DAG_{d}$, the semantic contribution of the disease term *t* to *d* is calculated below:

2 $$\begin{aligned} W_{1d}(t)&= \left\{ \begin{matrix} 1 &{} \text{ if } t = d \\ \text {max}\left\{ \varphi W_{1d}(t^{'}\mid t^{'}\ \ \text {is a child of}\ \ t ) \right\} &{} \text{ if } t\ne d \end{matrix}\right. \end{aligned}$$

where $\varphi$ represents the semantic contribution factor, which we assign a value of 0.5 following the work of Wang et al.^29^. The semantic score of disease *d* was computed as shown below:

3 $$\begin{aligned} S_{1}(d)&= \sum _{\alpha \in T(d)}W_{1d}(\alpha ) \end{aligned}$$

Building upon the premise that diseases with a greater overlap in their DAGs are likely to demonstrate higher similarity, the semantic similarity score between disease $d_{i}$ and disease $d_{j}$ were calculated as shown below:

4 $$\begin{aligned} DS_{1}(d_{i},d_{j} )&= \frac{ {\textstyle \sum _{t\in T(d_{i} )\cap T(d_{j} ) }(W_{1d_{i} }(t)+W_{1d_{j} }(t)) } }{S_{1}(d_{i})+S_{1}(d_{j}) } \end{aligned}$$

#### Disease semantic similarity 2

Due to the shortcomings of the semantic similarity measure presented by Wang et al.^29^, Chen et al.^26^ introduced an alternative measure. Specifically, the second semantic contribution score $W_{2d}$ for each disease *d* is described below:

5 $$\begin{aligned} W_{2d}&= - log\frac{\text {the number of DAGs including}\ d }{\text {the number of disease}} \end{aligned}$$

We utilized the second semantic contribution score $W_{2d}$ to compute the disease semantic score $S_{2}$ and semantic similarity $DS_{2}$ between $d_{i}$ and $d_{j}$. The specific formulas are illustrated below:

6 $$\begin{aligned} S_{2}(d)&= \sum _{\alpha \in T_{d} }W_{2d}(\alpha ) \end{aligned}$$

7 $$\begin{aligned} DS_{2}(d_{i},d_{j})&= \frac{ {\textstyle \sum _{d_{t}\in A_{d_{i} }\cap A_{d_{j} }}W_{2d_{i} }(d_{t} )+ W_{2d_{j} }(d_{t} ) } }{S_{2}(d_{i})+S_{2}(d_{j}) } \end{aligned}$$

#### Integrated semantic similarity of disease

Based on these two measures, we use a weighted average strategy for integration. The calculation equation is shown below:

8 $$\begin{aligned} DS(d_{i},d_{j})&= \frac{DS_{1} (d_{i},d_{j})+DS_{2}(d_{i},d_{j})}{2} \end{aligned}$$

### GIPK similarity for miRNA and disease

To enrich the similarity measures, we employed Gaussian kernels to compute the Gaussian interaction profile kernel (GIPK) similarity of miRNA/disease. Initially, we utilized the vector $MD(m_{i})$ to depict the interaction characteristic of miRNA $m_{i}$ by exploring its associations with various diseases. Similarly, the vector $MD(d_{i})$ was employed to indicate the interaction characteristic of disease $d_{i}$. The specific formula is shown below:

9 $$\begin{aligned} MGKS(m_{i} ,m_{j} )&= exp\left( -\lambda _{m}\left\| MD(m_{i})- MD(m_{j}) \right\| ^{2} \right) \end{aligned}$$

10 $$\begin{aligned} DGKS(d_{i} ,d_{j} )&= exp\left( -\lambda _{d}\left\| MD(d_{i})- MD(d_{j}) \right\| ^{2} \right) \end{aligned}$$

where $MGKS(m_{i},m_{j} )$ indicates the GIPK similarity between miRNA $m_{i}$ and $m_{j}$, and $DGKS(d_{i},d_{j} )$ denotes the GIPK similarity between disease $d_{i}$ and $d_{j}$. The adjustable parameters $\lambda _{m}$ and $\lambda _{d}$ are determined using the following equations:

11 $$\begin{aligned} \lambda _{m} = 1/ \frac{1}{nm} { \sum _{i = 1}^{nm}\left\| MD(m_{i}) \right\| ^{2} } \end{aligned}$$

12 $$\begin{aligned} \lambda _{d} = 1/ \frac{1}{nd} { \sum _{i = 1}^{nd}\left\| MD(d_{i}) \right\| ^{2} } \end{aligned}$$

### Integrated similarity for miRNA and disease

To enhance the accuracy of miRNA/disease similarity, we incorporated *MF* and *DS* with GIPK similarity, respectively. The ultimate miRNA similarity *MM* and disease similarity *DD* were obtained as shown below:

13 $$\begin{aligned} MM i, j&= \left\{ \begin{array}{ll} MF(m_{i}, m_{j}) &{} \text{ if } MF(m_{i}, m_{j}) \ne 0, \\ MGKS(m_{i}, m_{j}) &{} \text{ otherwise. } \end{array}\right. \end{aligned}$$

14 $$\begin{aligned} DD i, j&= \left\{ \begin{array}{ll} DS(d_{i}, d_{j}) &{} \text{ if } DS(d_{i}, d_{j}) \ne 0, \\ DGKS(d_{i}, d_{j}) &{} \text{ otherwise. } \end{array}\right. \end{aligned}$$

### Heterogeneous network construction

To efficiently utilize the available prior knowledge, we constructed a heterogeneous network. First, we introduced MM and DD into the heterogeneous network to improve the overall performance of EMCMDA. Second, we used the association matrix $A_{MD}$ to complete this miRNA-disease heterogeneous network. Finally, we defined the goal matrix *H* by utilizing this heterogeneous network.

15 $$\begin{aligned} H&= \begin{bmatrix} MM &{} A_{MD} \\ A_{MD}^{T} &{} DD \end{bmatrix} \end{aligned}$$

#### EMCMDA

The present MDA matrix inherently exhibits sparsity, featuring low-rank structures and containing a substantial amount of redundancy information that can be leveraged for data recovery and feature extraction. Minimizing nuclear norm methods are often employed to address low-rank matrix completion problems. The nuclear norm is defined as the summation of singular values within a matrix. It is employed to enforce the low-rank constraint on the matrix, thereby facilitating dimensionality reduction. Let’s consider the objective function *H* as a pre-defined low-rank or approximately low-rank matrix, and *X* as the low-rank matrix we aim to recover. The issue of minimizing the nuclear norm for *X* can be stated the following way:

16 $$\begin{aligned} \begin{aligned}{}&\min _{X}\Vert X\Vert _{*} \\ \end{aligned} \end{aligned}$$

where $\left\| X \right\| _{*}= { \sum _{i=1}^{min(nm,nd)}}\sigma _{i}(X)$ indicates the nuclear norm for *X*. Given the possibility of a substantial presence of “noisy” data within miRNA and disease datasets, it becomes imperative for MDA prediction models to exhibit a high degree of tolerance towards potential noise. Below, a comprehensive noise tolerance matrix model is presented:

17 $$\begin{aligned} \min _{X}\Vert X\Vert _{*} \ \ \text{ s.t. } \left\| P_{\Omega }(X)- P_{\Omega }(H) \right\| _{F}\le \varepsilon _{0} \end{aligned}$$

where $\varepsilon _{0}$ signifies the noise parameter, $\Omega$ denotes the set of all known associated index pairs (i,j) in *H* and $P_{\Omega }$ indicates the projection operator on $\Omega$.

18 $$\begin{aligned} (P_{\Omega }(X))_{ij}&= \begin{Bmatrix} X_{i j}&\text{ if } (\textrm{i}, \textrm{j}) \in \Omega \\ 0&\text{ otherwise } \end{Bmatrix} \end{aligned}$$

Although nuclear norm minimization is a viable method for predicting MDAs, it still exhibits certain limitations. The size of the singular value reflects the amount of information in the matrix, with larger values carrying the main information and smaller values containing smaller changes or noise. The standard nuclear norm treats each singular value identically, which greatly limits its ability to handle practical problems. Therefore, we proposed the matrix truncated schatten p-norm minimization method for MDA prediction. The truncated schatten p-norm treats different singular values differently and retains the first *r* larger singular values, ignoring small singular values. In addition, the *p*th power of the remaining singular values is summed. Mathematically, it can be expressed as $\left\| X \right\| _{r}^{p}=\sum _{i=r+1}^{nm+nd}\sigma _{i}^{p}(x)$. This fully takes into account the physical significance of the singular values and yields a superior solution. Therefore, the truncated schatten p-norm exhibits greater proximity to the rank than other rank relaxation norms.

Next, the important lemma of truncated schatten p-norm is introduced to facilitate the solution.

##### Lemma 1

(See^30^ and^31^) Consider a matrix $X\in R^{(nm+nd)\times (nm+nd)}$ with a rank $s(s\le nm+nd)$, and its singular value decomposition as $X=U\bigtriangleup V^{T}$, where $U \in R^{(nm+nd)\times (nm+nd)}$ , $\bigtriangleup \in R^{(nm+nd)\times (nm+nd)}$, $V \in R^{(nm+nd)\times (nm+nd)}$. When $A \in R^{r\times (nm+nd)}$, $B \in R^{r\times (nm+nd)}$ and $0< p\le 1$, the optimization problem has optimal solution. The specific formula is shown below:

19 $$\begin{aligned} \Vert X\Vert _{\textrm{r}}^{p} = \min _{A,B} \sum _{i= 1}^{nm+nd}\left( 1-\sigma _{i}\left( B^{T} A\right) \right) \left( \sigma _{i}(X)\right) ^{p} \nonumber \\ \mathrm { s.t. } A A^{T} = I_{r \times r}, B B^{T} = I_{r \times r} \end{aligned}$$

Thanks to Lemma 1, we enhanced the initial model for minimizing the nuclear norm [Eq. (17)] and developed a new model:

20 $$\begin{aligned}&\min _{X} \sum _{i = 1}^{nm+nd}\left( 1-\sigma _{i}\left( B^{\top } A\right) \right) \left( \sigma _{i}(X)\right) ^{p}&\nonumber \\&\text{ s.t. } \left\| P_{\Omega }(X)- P_{\Omega }(H) \right\| _{F}\le \varepsilon _{0} ,\ \nonumber \\ \ A \in {R}^{r\times (nm+nd)}, B \in {R}^{r\times (nm+nd)},\ \ {}&\nonumber \\&A A^{\top } = I_{r \times r},\ B B^{\top } = I_{r \times r} , \ \textrm{and} \quad 0<p \le 1&\end{aligned}$$

Equation (20) is non-convex, providing a more accurate approximation than the convex nuclear norm. However, its solution poses a challenge, as conventional methods are inadequate for addressing this non-convexity. For this reason, we first transformed the model [Eq. (20)].

We let $Q(\sigma (X)) = \sum _{i = 1}^{nm+nd}(1-\sigma _{i}(B^{T}A)(\sigma _{i}(X))^{p}$. Subsequently, we computed the derivative of the equation with regard to $\sigma (X)$.

21 $$\begin{aligned} \bigtriangledown Q(\sigma (X)) = \sum _{i = 1}^{nm+nd}p(1-\sigma _{i}(B^{T}A)(\sigma _{i}(X))^{p-1} \end{aligned}$$

Then, the first-order Taylor expansion for $Q(\sigma (X))$ was attained as shown below:

22 $$\begin{aligned} \begin{aligned} Q(\sigma (X))&= Q\left( \sigma \left( X_{k}\right) \right) +\left\langle \nabla Q\left( \sigma \left( X_{k}\right) \right) , \sigma (X)-\sigma \left( X_{k}\right) \right\rangle \\&= \nabla Q\left( \sigma \left( X_{k}\right) \right) \cdot \sigma (X) \\&= \sum _{\textrm{i} = 1}^{nm+nd} \textrm{p}\left( 1-\sigma _{\textrm{i}}\left( B^{T} A\right) \right) \left( \sigma _{i}\left( X_{k}\right) \right) ^{p-1} \cdot \sigma _{i}(X) \end{aligned} \end{aligned}$$

We let $\omega _{i}=p(1-\sigma _{i}\left( B^{T}A) \right) \left( \sigma _{i}(X_{k} ) \right) ^{p-1}$. Then $Q\left( \sigma (x) \right) = {\textstyle \sum _{i=1}^{nm+nd}\omega _{i}\sigma _{i}(X)}$, where $W:= \left\{ \omega _{i} \right\} _{1}^{nm+nd}$ is a weight sequence. After processing, we acquired the following solvable convex optimization model:

23 $$\begin{aligned} \min _{\textrm{X}} \sum _{i = 1}^{nm+nd} \omega _{i} \sigma _{i} \text{ s.t. } \left\| P_{\Omega }(X)- P_{\Omega }(H) \right\| _{F}\le \varepsilon _{0} \end{aligned}$$

However, solving models with inequality constraints presents numerous challenges. Therefore, it is a widely adopted approach to replace the constrained model with a regularized counterpart. The incorporation of soft regularization not only allows for the accommodation of unforeseen noise but also significantly enhances the efficiency of our problem-solving procedures. Furthermore, we applied a constraint within the range of [0, 1] to all matrix values to ensure their practical significance^32,33^. In conclusion, we constructed the following model:

24 $$\begin{aligned} \min _{X} \sum _{i = 1}^{nm+nd} \omega _{i} \sigma _{i}+\frac{\alpha }{2}\left\| P_{\Omega }(X)-P_{\Omega }(H)\right\| _{F}^{2} \nonumber \\ \text{ s.t. } 0 \le X_{i j} \le 1(0 \le i, j \le nm+nd) \end{aligned}$$

where $\alpha$ represents a equilibrium coefficient and $0\le X_{i,j}\le 1$ (where $0\le i,j\le nm+nd$) signifies that all the elements in matrix *X* fall within the range of [0, 1].

We formulated a framework utilizing the alternating direction multiplier method (ADMM)^34^ to handle the optimization issue as shown below.

**Step 1**: Initialize $X_{1} =H$ and calculate the (*l*+1)-th iteration of $X_{l}=U_{l}\bigtriangleup _{l}V_{l}^{T}$. Next, determine $A_{l}$ and $B_{l}$ based on the values of $U_{l}$ and $V_{l}$. Experimental validation shows that $l\in [1,4]$ gives the optimal result.

**Step 2**: Calculate the *k*-th iteration of $W= \left\{ \omega _{i} \right\} _{1}^{nm+nd}$. Following this, the ADMM-based framework is employed for solving equation (24). Experimental validation shows that $k=1$ produces the best result.

To facilitate the computation, we introduce an auxiliary matrix *T* for subsequent solution.

25 $$\begin{aligned}&\min _{X} \sum _{i= 1}^{nm+nd} \varpi _{i} \sigma _{i}+\frac{\alpha }{2}\left\| P_{\Omega }(X)-P_{\Omega }(H)\right\| _{F}^{2} \nonumber \\&\text{ s.t. } \textrm{X} = \textrm{T},\ 0 \le X_{i j} \le 1(0 \le i, j \le nm,nd) \end{aligned}$$

The augmented Lagrangian form of Eq. (25) is represented below:

26 $$\begin{aligned} \ell (T, X, E, \alpha , \beta ) = \sum _{i = 1}^{nm+nd} \varpi _{\textrm{i}}\nonumber \\ \sigma _{i}+\frac{\alpha }{2}\left\| P_{\Omega }(T)-P_{\Omega }(H)\right\| _{F}^{2}\nonumber \\ +{\text {Tr}}\left( \textrm{E}^{\textrm{T}}(X-T)\right) +\frac{\beta }{2}\Vert X-T\Vert _{F}^{2} \end{aligned}$$

where *E* denotes the Lagrange multiplier, $\beta$ denotes the penalty parameter. The minimization of Eq. (26) is an iterative computation process. In the *k*-th iteration step, $T_{k+1}$, $X_{k+1}$, and $E_{k+1}$ are calculated serially. The following is the detailed procedure for the iterative algorithm’s solution process.

**Update **$T_{k+1}$: Fix $X_{k}$ and $E_{k}$ to update $T_{k+1}$ via minimizing function $\ell$(*T*,*X*,*E*,$\alpha$,$\beta$).

27 $$\begin{aligned} \begin{aligned} T_{k+1} = \underset{0 \le T_{ij} \le 1}{\arg {\text {min}}\ell }\left( T,X_{k}, E_{k},\alpha ,\beta \right) \\ = \underset{0 \le T_{ij} \le 1}{\arg \min } \frac{\alpha }{2}\left\| P_{\Omega }(T)-P_{\Omega }(H)\right\| _{F}^{2} \\ +{\text {Tr}}\left( E_{k}^{T}\left( X_{k}-T\right) \right) +\frac{\beta }{2}\left\| X_{k}-T\right\| _{F}^{2} \end{aligned} \end{aligned}$$

We attain the optimal solution $\overline{T}_{k+1}$ of Eq. (27) exclusively when the derivative of Eq. (27) is 0, as shown below:

28 $$\begin{aligned} \alpha P_{\Omega }^{*}(P_{\Omega }(\overline{T}_{k+1})-P_{\Omega }(H))-Z_{k}-\beta (X_{k}-\overline{T}_{k+1})&= 0 \end{aligned}$$

where $P_{\Omega }^{*}$ represents the adjoint operator of $P_{\Omega }$, and it fulfills the condition $P_{\Omega }^{*}P_{\Omega }=P_{\Omega }$. The solution is continued as follows:

29 $$\begin{aligned}&\overline{T}_{k+1} = \left( I+\frac{\alpha }{\beta } P_{\Omega }^{*} P_{\Omega }\right) ^{-1}\left( \frac{1}{\beta } E_{k}+\frac{\alpha }{\beta } P_{\Omega }^{*} P_{\Omega }(H)+X_{k}\right)&\nonumber \\ {}&= \left( I-\frac{\alpha }{\alpha +\beta } P_{\Omega }^{*} P_{\Omega }\right) \left( \frac{1}{\beta } E_{k}+\frac{\alpha }{\beta } P_{\Omega }^{*} P_{\Omega }(H)+X_{k}\right)&\nonumber \\&= \left( \frac{1}{\beta } E_{k}+\frac{\alpha }{\beta } P_{\Omega }(H)+X_{k}\right) -\frac{\alpha }{\alpha +\beta }P_{\Omega }\left( \frac{1}{\beta } E_{k}+\frac{\alpha }{\beta } P_{\Omega }(H)+X_{k}\right)&\end{aligned}$$

where *I* denotes the identity operator. Based on reference^35^, it is known that $(I+\frac{\alpha }{\beta }P_{\Omega }^{*}P_{\Omega })^{-1}=(I-\frac{\alpha }{\alpha +\beta }P_{\Omega }^{*}P_{\Omega })$. To ensure that the predictions are meaningful, we restrict the elements of $\overline{T}_{k+1}$ to the range [0, 1].

30 $$\begin{aligned} \left[ T_{\textrm{k}+1}\right] _{i j}&= \begin{Bmatrix} 0&\text{ if } \overline{T}_{k+1_{ij}}<0 \\ \overline{T}_{k+1}&\text{ if } 0 \le \overline{T}_{k+1_{ij}} \le 1 \\ 1&\text{ if } \overline{T}_{k+1_{ij}}>1 \end{Bmatrix}. \end{aligned}$$

**Update **$X_{k+1}$: Fix $T_{k+1}$ and $E_{k}$ to update $X_{k+1}$ by minimizing function $\ell$(*T*,*X*,*E*,$\alpha$,$\beta$) .

31 $$\begin{aligned}&X_{\textrm{k}+1} = \underset{X}{\arg \min }\ \mathrm {\ell } \left( T_{k+1},X,E_{k},\alpha , \beta \right)&\nonumber \\&= \underset{X}{{\text {argmin}}} \sum _{i= 1}^{nm+nd} \omega _{i} \sigma _{i}+{\text {Tr}}\left( E_{\textrm{k}}^{\textrm{T}}\left( X-T_{k+1}\right) \right) +\frac{\beta }{2}\left\| X-T_{k+1}\right\| _{F}^{2}&\nonumber \\ \quad&= \underset{X}{{\text {argmin}}} \sum _{i = 1}^{nm+nd} \omega _{i} \sigma _{i}+\frac{\beta }{2}\left\| X-\left( T_{k+1}-\frac{1}{\beta } E_{k}\right) \right\| _{F}^{2} = S_{\omega ,\frac{1}{\beta }}(Q_{X} )&\end{aligned}$$

$S_{\omega ,\frac{1}{\beta } }(Q):=Umax(\Delta -\frac{1}{\beta } diag(W),0)V^{T}$, where $S{\omega ,\frac{1}{\beta } }(\cdot )$ is the weighted singular value contraction operator and $W= \left\{ \omega _{i} \right\} _{1}^{nm+nd}$ (refer to^36^). **Update **$E_{k+1}$: Fix $T_{k+1}$ and $X_{k+1}$ to update $E_{k+1}$.

32 $$\begin{aligned} E_{\textrm{k}+1}&= E_{k}+\frac{\partial L(T, X, E, \alpha , \beta )}{\partial E} \nonumber \\&= E_{k}+\beta \left( X_{k+1}-T_{k+1}\right) \end{aligned}$$

Keep iterating according to the above update rule until the convergence conditions $$\textrm{S1}_{k+1} = \frac{\left\| X_{k+1}-X_{k}\right\| _{F}}{\left\| X_{k}\right\| _{F}} \le \varepsilon _{1}$$ and $$\textrm{S} 2_{k+1} = \frac{\left| S 1_{k+1}-S 1_{k}\right| }{\max \left\{ \left| S 1_{k}\right| , 1\right\} } \le \varepsilon _{2}$$ are satisfied. Here, the values of $$\varepsilon _{1}$$ and $$\varepsilon _{2}$$ refer to the paper by Yang et al.^37^. The complemented adjacency matrix $$H^{*} $$ is shown below:

33 $$\begin{aligned} H^{*} = \left[ \begin{array}{cc} \textrm{MM}^{*} &{} \textrm{A}_{\textrm{MD}}^{*} \\ \mathrm {~A}_{\textrm{MD}}^{\mathrm {T*}} &{} \textrm{DD}^{*} \end{array}\right] \end{aligned}$$

We fetched the complemented MDA matrix $A_{MD}^{*}$ from $H^{*}$. Specifically, we replaced all the unrecorded values in $A_{MD}^{*}$ with predicted scores within the [0, 1] range, indicating the probability of potential MDAs. To elucidate this solution procedure, we present 1 below.

**Algorithm 1:**
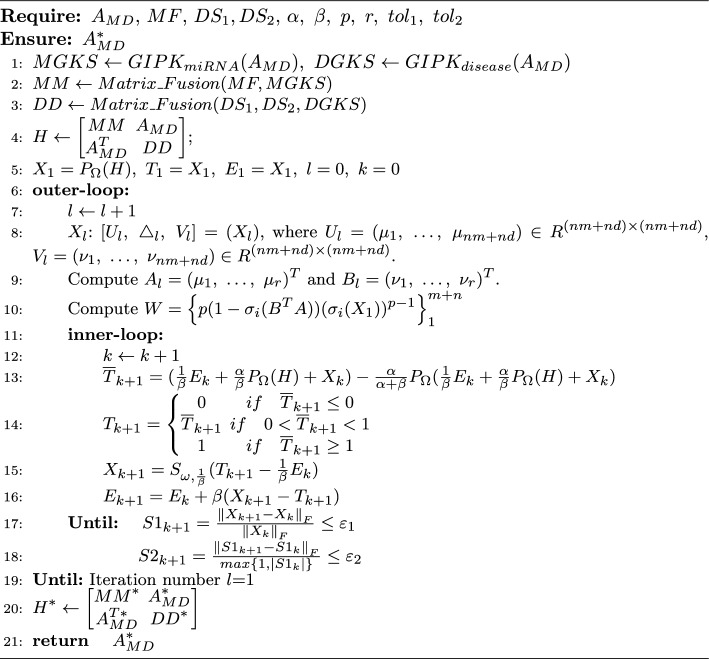
**EMCMDA algorithm.**

## Results

### Performance evaluation

In this study, the predictive capability of EMCMDA is assessed through Global LOOCV and 5-fold CV using the benchmark dataset. To assess the proposed model, we compared its predictions with those generated by HGCLAMIR^16^, BNNRMDA^24^, WBNPMD^19^, KATZBNRA^18^, PMFMDA^25^, IMCMDA^26^.

**Figure 2:**
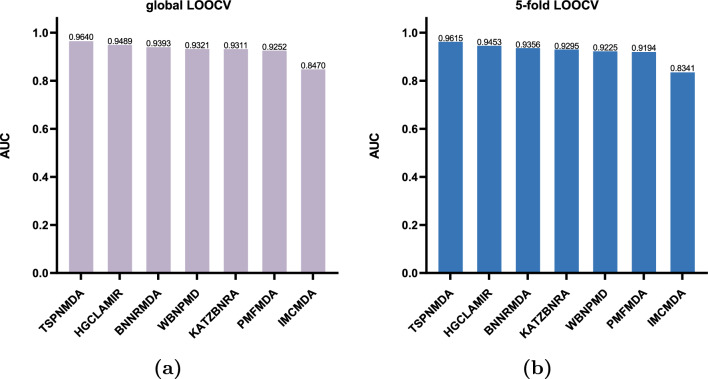
Global LOOCV and 5-fold CV were employed on the benchmark dataset to compare the predictive capabilities of various models.

**Figure 3:**
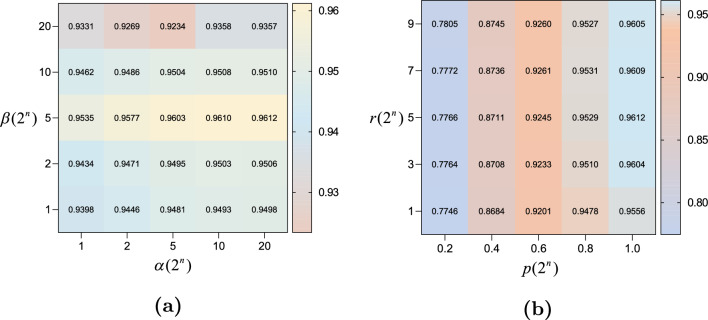
The results of the prametric sensitivity analysis.

#### Global LOOCV

To make the most of the existing biological data, we utilized Global LOOCV on the benchmark dataset. In Global LOOCV, we systematically treated each of the 5430 known MDAs as a test set, while the remainder of the known associations were employed as training. All unidentified MDA pairs were employed as candidate set. After EMCMDA computes all relevant prediction scores, we ranked these scores in descending order for both the test and candidate samples. Finally, we employed distinct thresholds to compute AUC. As depicted in Fig. 2a, EMCMDA got the highest AUC (0.9640). It also demonstrates that EMCMDA outperforms other comparative methods in the study.

#### 5-fold CV

The 5-fold CV was implemented to further validate EMCMDA’s prediction performance. In 5-fold CV, all known MDAs were split into five equal-sized subsets. For each fold, a segment was designated as the testing set, and the other four segments were used for training purposes. We performed the same operation in the other comparison models. As with Global LOOCV, we used AUC values to compare these models’s performance. As depicted in Fig. 2b, EMCMDA obtained the highest AUC (0.9615). This also demonstrates the superior ability of our model to predict potential MDAs.

### Parametric sensitivity analysis

A sensitivity analysis of the important parameters of the model was performed to ensure that EMCMDA achieved better prediction. The following parameters are our main focus: equilibrium coefficient $\alpha$, penalty parameter $\beta$, power of singular values *p* and truncation position of the target matrix rank *r*. We implemented 5-fold CV on the benchmark dataset to determine the optimal parameters of EMCMDA. The results are depicted in Fig. 3. The AUC values was utilized as an indicator for the evaluation of the parameter. We first optimized the values of $\alpha$ and $\beta$ and subsequently held them constant while determining the optimal values for *p* and *r*. As illustrated in Fig. 3, the model achieved the highest AUC (0.9612) when $\alpha$=20, $\beta$=5, *p*=1 and *r*=5. Based on the above, we here set $\alpha$=20, $\beta$=5, *p*=1 and *r*=5.

### Experimental results on HDMM v3.0

To assess the EMCMDA’s applicability on different datasets, we conducted Global LOOCV and 5-fold CV based on the HMDD v3.0 database^38^. We acquired 1062 miRNAs, 893 diseases and 35362 known MDAs from the HMDD v3.0 database. In this context, we set the parameters $\alpha$=2, $\beta$=2, *p*=1 and *r*=3. Table 1 lists the AUC scores for both HMDD v2.0 and HMDD v3.0 datasets. In the global LOOCV, EMCMDA achieves AUC scores of 0.9640 for HMDD v2.0 and 0.9725 for HMDD v3.0. Meanwhile, in the 5-fold CV, EMCMDA demonstratesAUC scores of 0.9615 for HMDD v2.0 and 0.9706 for HMDD v3.0. It is evident from the table that EMCMDA continues to exhibit excellent performance when applied to the newly collected dataset, reaffirming its robustness and effectiveness in diverse data settings.

**Table 1:** Performance comparison of EMCMDA using AUC values on two datasets.

Experiments	HMDD v2.0	HMDD v3.0
Global LOOCV	0.9640	0.9725
5-fold CV	0.9615	0.9706

### Ablation experiment

To verify the importance of GIPK similarity, we presented a variant of EMCMDA that does not contain a GIPK similarity method (EMCMDA-W). Based on implementing 5-fold CV on the benchmark dataset, we compared the performance of both using the AUC and AUPR metrics. As illustrated in Table 2, EMCMDA attains an AUC of 0.9615 and an AUPR of 0.3279, while EMCMDA-W achieves an AUC of 0.9036 and an AUPR of 0.2095. The AUC and AUPR scores for EMCMDA are higher than those of EMCMDA-W under different metrics. Therefore, we can assert that GIPK similarity plays a substantial role in enhancing the predictive power of EMCMDA.

**Table 2:** The result of the ablation experiment.

Models	AUC	AUPR
EMCMDA	0.9615	0.3279
EMCMDA-W	0.9036	0.2095

### Sensitivity analysis with known number of associations

**Figure 4:**
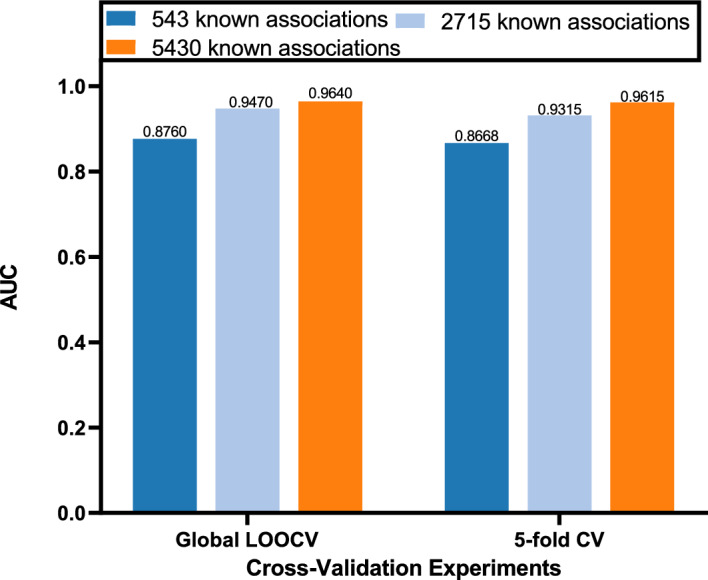
The result of sparse matrix sensitivity analysis.

To examine the impact of the quantity of known associations on the model’s performance, we randomly selected 10% and 50% of the original 5430 known associations to construct the new association matrix. We executed Global LOOCV and 5-fold CV to assess EMCMDA using the benchmark dataset. The results are depicted in Fig. 4. In the global LOOCV, EMCMDA achieves AUC scores of 0.8760, 0.9470, and 0.9640, corresponding to 10%, 50%, and 100% of the original known associations, respectively. In the 5-fold CV, EMCMDA demonstrates AUC scores of 0.8668, 0.9315, and 0.9615, respectively. Figure 4 vividly illustrates the trend of increasing AUC values for EMCMDA as the number of known associations grows. Therefore, it can be inferred that the predictive capability of EMCMDA shows a positive correlation with the quantity of known associations.

### Hypothesis testing

We employed hypothesis testing to analyze the disparity in predictive capabilities between EMCMDA and other previously employed models. Initially, we assumed that the results obtained from Global LOOCV and 5-fold CV were equivalent between EMCMDA and the comparison models. Subsequently, we conducted t-tests separately on the two CV results for EMCMDA and the other comparison models. The p-values resulting from these hypothesis tests are presented in Table 3. The significant differences between EMCMDA and other comparison methods (BNNRMDA, WBNPMD, KATZBNRA, PMFMDA and IMCMDA) can be observed. Given that the obtained p-value between our method and the compared models is substantially less than 0.05, we can confidently assert that EMCMDA exhibits significant distinctions and outperforms other comparison models.

**Table 3:** P-value derived from hypothesis testing by EMCMDA and other comparative methods.

Methods	Global LOOCV	5-fold CV
BNNRMDA	2.88023e-92	6.2241e−159
WBNPMD	5.6165e−149	6.0923e−177
KATZBNRA	9.5746e−158	2.9365e−168
PMFMDA	1.9862e−213	2.7323e−180
IMCMDA	0	2.1194e-228

### Performance evaluation of multiple metrics

**Figure 5:**
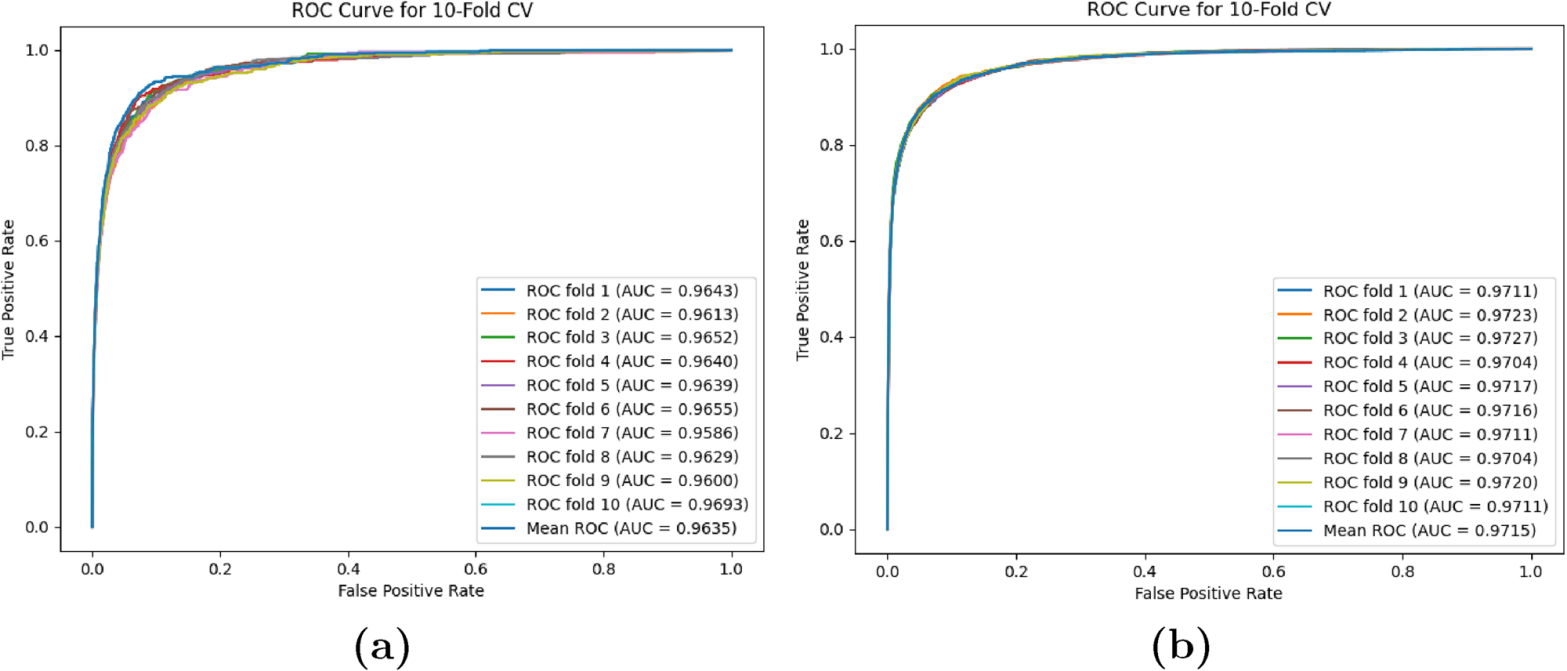
(**a**) ISIMC conducts 10-fold CV on the HMDD v2.0 dataset; (**b**) ISIMC conducts 10-fold CV on the HMDDv3.0 dataset.

To adequately assess the EMCMDA’s reliability, we conducted 10-fold CV on the HMDD v2.0 and HMDDv3.0 datasets. As depicted in Fig. 5, EMCMDA obtained AUC values of 0.9635 and 0.9715 on the respective datasets, underscoring its reliability in MDA prediction. Additionally, we introduced five supplementary metrics to comprehensively assess the EMCMDA’s performance. To maintain a balance between positive and negative samples, we randomly selected negative samples from the unknown MDAs while ensuring a 1:1 ratio between the number of positive and negative samples. Subsequently, these metrics were computed based on three thresholds that optimize Accuracy, F1 Score, and MCC. Table 4 showcases that EMCMDA acquired Accurary of 0.9341, Precision of 0.8229, Recall of 0.8155, F1 score of 0.7961, and MCC of 0.7576, affirming EMCMDA is an excellent MDA prediction model.

**Table 4:** Five additional metrics were incorporated to validate the EMCMDA’s efficacy.

Thresholds	Accurary	Precision	Recall	F1 score	MCC
$T_{1}$	0.9341	0.8229	0.7716	0.7961	0.7576
$T_{2}$	0.9307	0.7897	0.7976	0.7933	0.7519
$T_{3}$	0.9301	0.7768	0.8155	0.7953	0.7538

Notes: $T_{1}$, $T_{2}$, and $T_{3}$ denote the threshold values that specifically maximize optimize the accuracy, F1 score, and MCC respectively.

**Table 5:** We predicted the top 50 miRNAs for lung tumors (i and ii refer to dbDEMC and miRCancer, respectively).

miRNA(1-25)	Evidence	miRNA(26-50)	Evidence
hsa-mir-320d	i	hsa-mir-527	i
hsa-mir-320b	i	hsa-mir-522	i
hsa-mir-320e	i	hsa-mir-517c	i
hsa-mir-450a	i	hsa-mir-92	i
hsa-mir-450b	i	hsa-mir-320c	i
hsa-mir-1293	i	hsa-mir-608	i
hsa-mir-202	i	hsa-mir-500a	i
hsa-mir-1245a	i	hsa-mir-1303	i
hsa-mir-1245b	i	hsa-mir-2110	i
hsa-mir-1323	i	hsa-mir-1915	i
hsa-mir-1469	i,ii	hsa-mir-612	i
hsa-mir-181	Unconfirmed	hsa-mir-657	i
hsa-mir-2355	i	hsa-mir-519e	i
hsa-mir-3130	i	hsa-mir-499b	i
hsa-mir-3186	i	hsa-mir-147a	i
hsa-mir-4257	i	hsa-mir-632	i
hsa-mir-4306	i	hsa-mir-922	i
hsa-mir-718	i	hsa-mir-1471	Unconfirmed
hsa-mir-371	i	hsa-mir-505	i
hsa-mir-1202	i	hsa-mir-526b	i
hsa-mir-1231	i	hsa-mir-105	i
hsa-mir-1234	i	hsa-mir-200	Unconfirmed
hsa-mir-1301	i	hsa-mir-1258	i,ii
hsa-mir-26	Unconfirmed	hsa-mir-1249	i
hsa-mir-500b	i	hsa-mir-526a	i

**Table 6:** We predicted the top 50 miRNAs for breast tumors (i and ii refer to dbDEMC and miRCancer, respectively).

miRNA(1-25)	Evidence	miRNA(26-50)	Evidence
hsa-mir-31	i	hsa-mir-25	i,ii
hsa-mir-135a	i	hsa-mir-497	i,ii
hsa-mir-106a	i,ii	hsa-mir-148b	i,ii
hsa-mir-135b	i	hsa-mir-129	i,ii
hsa-mir-451a	i,ii	hsa-mir-34a	i,ii
hsa-mir-421	i,ii	hsa-mir-20a	i,ii
hsa-mir-191	i,ii	hsa-mir-132	i,ii
hsa-mir-183	i,ii	hsa-mir-20b	i,ii
hsa-mir-137	i	hsa-mir-150	i,ii
hsa-mir-181b	i,ii	hsa-mir-93	i,ii
hsa-mir-498	i,ii	hsa-mir-92b	i
hsa-mir-658	i	hsa-mir-24	i,ii
hsa-mir-340	i,ii	hsa-mir-17	i,ii
hsa-mir-214	i,ii	hsa-mir-34c	i,ii
hsa-mir-18a	i,ii	hsa-mir-34b	i,ii
hsa-mir-18b	i,ii	hsa-mir-101	i,ii
hsa-mir-133b	i,ii	hsa-mir-203	i,ii
hsa-mir-10b	i,ii	hsa-let-7g	i,ii
hsa-mir-124	i,ii	hsa-mir-184	i
hsa-mir-107	i,ii	hsa-let-7f	i
hsa-mir-27a	i,ii	hsa-mir-424	i
hsa-mir-139	i,ii	hsa-mir-148a	i,ii
hsa-mir-141	i,ii	hsa-let-7i	i,ii
hsa-mir-153	i,ii	hsa-mir-192	i
hsa-mir-19a	i,ii	hsa-let-7d	i,ii

**Figure 6:**
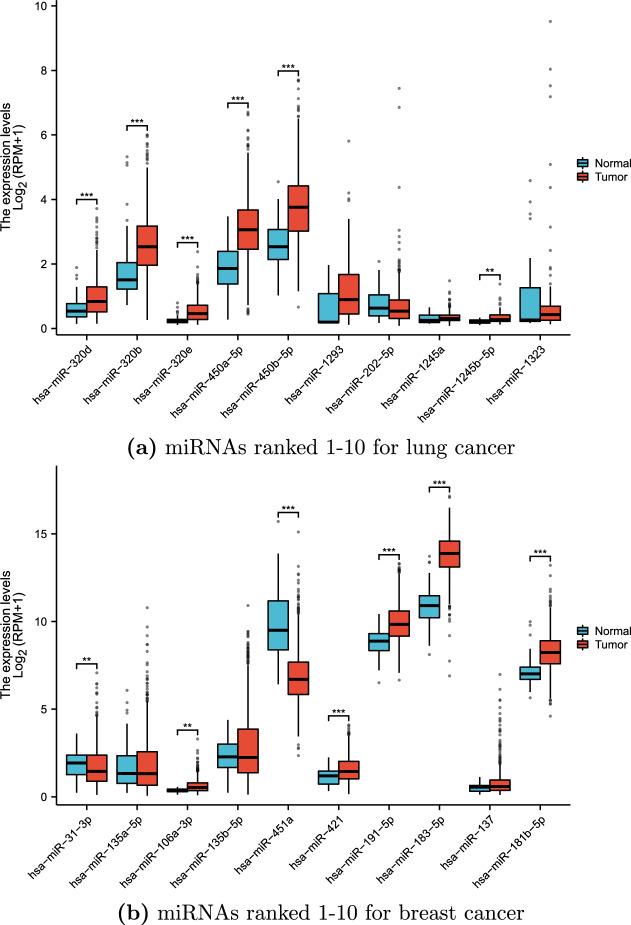
The outcomes of the differential expression analysis for miRNAs.

## Case studies

We tested two common human diseases (lung tumors and breast tumors) to demonstrate the ability of EMCMDA for practical applications. The EMCMDA model was trained using data sourced from the HMDD v2.0 database. For both lung and breast tumours, we have designated certain disease-associated miRNAs both as unknown associations, effectively treating them as novel diseases. For each disease under investigation, candidate miRNAs were sorted according to their predicting correlation scores. The top 50 candidates were subsequently authenticated using two other well-established MDA datasets, namely dbDEMC^39^ and miR2Cancer^40^. In all case studies, a significant quantity of disease-associated miRNAs were validated through experimental evidence, underscoring the reliability of EMCMDA’s predictions.

Lung tumors are widely recognized as one of the deadliest and most challenging cancers to treat due to their tendency to spread or metastasize early in their development. The lungs are particularly vulnerable to tumor metastasis in other parts of the body^41^. Recent biological experiments have provided strong evidence of miRNAs related to lung tumors. For example, miR-718 has demonstrated its efficacy in hindering the advancement of non-small cell lung cancer (NSCLC) by targeting CCNB1 mRNA as a therapeutic intervention^42^. Moreover, a notable upsurge in miR-522 expression was observed in human tissues affected by NSCLC. Inhibiting miR-522 has shown to be an effective strategy in restraining NSCLC cell proliferation and inducing apoptosis^43^. Moreover, the introduction of exogenous miR-202 has been demonstrated to reduce NSCLC cell viability, migration, and invasion^44^. Notably, the outcomes reveal that 46 of the top 50 predicted miRNAs linked to lung tumors were validated in either the dbDEMC or miR2Cancer datasets (see Table 5).

Breast tumors are among the most common cancers affecting women. However, the rates of cure and prognosis can be significantly improved through early detection, regular screening, and timely treatment^45^. An increasing number of biological experiments has affirmed the effect of miRNAs in breast tumors. For example, miR-132 assumes a crucial function in restraining the proliferation, invasion, migration, and metastasis of breast cancer through direct inhibition of HN1^46^. Additionally, miR-34a suppresses the proliferation of breast cancer via specifically targeting LMTK3 and holds promise as an anti-ER (estrogen receptor) agent in breast cancer therapy^47^. Moreover, Upregulation of miR-101 effectively suppresses the development of breast cancer cells^48^. Notably, the results indicate that all of the top 50 predicted miRNAs linked to breast tumors were certified in either the dbDEMC or miR2Cancer datasets (refer to Table 6).

Furthermore, we acquired miRNAseq data associated with lung and breast cancers, enabling us to perform a comparative analysis of the differential expression patterns of the top 10 miRNAs predicted by EMCMDA for these specific diseases. Notably, EMCMDA’s predictions regarding these miRNAs were validated through expression changes observed in expression within the corresponding disease contexts. This supplementary evidence serves to further validate the efficacy of our model. Figure 6 exhibits the detailed outcomes of the differential expression analysis.

## Discussion and conclusion

As our comprehension of the fundamental biological mechanisms underlying various diseases continues to grow, the implications of MDA prediction are poised to be both extensive and profound. This endeavor is expected not only to significantly enhance our ability to detect diseases in their early stages but also to advance our strategies for addressing complex diseases. In the last few years, more and more computational models have been developed. HGCLAMIR^16^ combines view-aware attention mechanisms of hypergraph contrast learning and combined multi-view representation techniques to forecast MDAs. Its advantage lies in proposing a multi-view representation integration approach, enriching embedded representation information. However, it lacks interpretability. BNNRMDA^24^ employs bounded kernel paradigm regularization for predicting potential MDAs. Its innovation lies in constraining the prediction structure to the interval of 0-1, ensuring interpretability of predictions. Nonetheless, the model’s solution is suboptimal. PMFMDA^25^ uses probability matrix decomposition to predict unknown MDAs. However, it relies on a single similarity measure and its solution is suboptimal. Current MDA prediction models fail to sufficiently capture the miRNA/disease similarities. While matrix completion proves effective for association prediction, existing models fall short in delivering optimal solutions. To address these challenges, we introduce the EMCMDA model to address the issue of missing MDAs by minimizing matrix truncated schatten p-norm. The key contributions of the EMCMDA model are outlined below: (i) We calculated the similarities across multiple sources for miRNA/disease pairs and combined this information to create a holistic miRNA/disease similarity measure. This enriches the similarity types, reduces the bias caused by a single similarity, and improves the similarity accuracy of miRNAs/diseases. (ii) We complement the predicted values of the unknown MDAs by minimizing matrix truncated schatten p-norm. This norm offers a more accurate approximation to the rank than other rank relaxation norms, and therefore obtains more accurate solutions. (iii) We improved the conventional singular value contraction algorithm through using a weighted singular value contraction technique. This technique dynamically adjusts the degree of contraction using the significance of each singular value, ensuring that the physical meaning of these singular values is fully considered.

We conducted Global LOOCV and 5-fold CV using the benchmark dataset, and EMCMDA consistently achieved the highest AUC values, surpassing the AUC of all compared methods. When applied to the HMDD v3.0 dataset, EMCMDA yielded AUCs of 0.9756 and 0.9706 for Global LOOCV and 5-fold CV, respectively. These results demonstrate the robust generalization capability of EMCMDA across different datasets. To further illustrate the practical utility of EMCMDA, we conducted two case studies that highlight its efficiency in real-world applications.

While EMCMDA demonstrates strong predictive performance, it does come with certain limitations. First, the model’s parameters may not always be optimized, potentially affecting prediction accuracy. Second, the utilization of a weighted average strategy for merging multi-source data pertaining to miRNAs and diseases may not represent the most optimal fusion method. Third, the available correlation information remains limited, thereby constraining the predictive capacity of the model. Lastly, although our model can predict potential MDAs, it falls short in pinpointing the specific mechanisms through which miRNAs contribute to disease onset. The study of gene/protein signaling networks using ode-based theoretical models is not only crucial for identifying potential therapeutic targets for diseases, but also helps to explore the mechanisms of gene/protein signaling networks in disease treatment^49,50^. Therefore, we can achieve a more comprehensive prediction by integrating the miRNA expression regulation information obtained from the ODE-based theoretical model into the heterogeneous network. Addressing these challenges is a key component of our future research.

## Data Availability

The datasets related to this project can be accessed for download at https://github.com/Normalqq/EMCMDA.git.

## Code availability

The code for this work can be downloaded at https://github.com/Normalqq/EMCMDA.git.
